# SHIVs, monkeys, and anti-HIV-1 neutralizing antibodies

**DOI:** 10.1186/1742-4690-10-S1-O41

**Published:** 2013-09-19

**Authors:** Malcolm A Martin

**Affiliations:** 1 National Institute of Allergy and Infectious Diseases, National Institutes of Health, Bethesda, Maryland 20892, USA

## 

One of the major obstacles in developing an effective HIV vaccine has been the inability to design immunogens capable of eliciting broadly reacting neutralizing antibodies (NAbs). We have previously described the construction and properties of the pathogenic R5-tropic SHIV_AD8_, which expresses the envelope glycoprotein derived from HIV-1_Ada_ strain and exhibits a Tier 2 neutralization phenotype. During its characterization, we discovered that SHIV_AD8_ had unique properties: nearly all infected rhesus monkeys generated broadly reacting NAbs capable of blocking the infection of Tier 1 HIV-1 isolates. Furthermore, approximately 25% of infected animals developed Nabs against Tier 2 HIV-1 strains, and one infected macaque was an “elite neutralizer”, producing potent, cross-clade NAbs that targeted the gp120 N332 glycan. We have used the SHIV_AD8_ macaque system as an animal model to investigate the steps associated with the production of broadly reacting anti-HIV-1 NAbs including the role of follicular helper T cells. In a final group of experiments, the role of new potent, broadly acting, anti-HIV monoclonal NAbs to block virus acquisition or as immunotherapy in chronically infected animals will be presented.

